# Kernel Fatty Oils of *Cinnamomum chago* Alleviate Functional Constipation by Improving Gut Microbiota and Intestinal Barrier Function

**DOI:** 10.3390/nu18182979

**Published:** 2026-09-11

**Authors:** Senye Wang, Yilin Zhu, Jing Han, Suxing Tu, Mengting Li, Ruiyang Gao, Ziheng Jin, Zhenhua Liang, Yu Zhang, Wenyi Kang

**Affiliations:** 1 National R & D Center for Edible Fungus Processing Technology, Henan University, Kaifeng 475004, China; wangsenye96@163.com (S.W.); zhuyilin0208@163.com (Y.Z.); h042163780@163.com (J.H.); 15339031016@163.com (S.T.); 15100136694@163.com (M.L.); 15538621776@163.com (R.G.); 2 College of Agriculture, Henan University, Kaifeng 475004, China; 3 School of Life Sciences, Henan University, Kaifeng 475004, China; 4 Henan Key University-Enterprise R&D Center for Special Foods, Kaifeng 475004, China; jinzh@hazhongda.com; 5 Key Laboratory for Innovation and Manufacturing of Specialty Plant Health Foods in Henan Province, Kaifeng 475004, China; 6 State Key Laboratory of Phytochemistry and Natural Medicines, Kunming Institute of Botany, Chinese Academy of Sciences, Kunming 650201, China; 7 Yunnan Key Laboratory for Integrative Conservation of Plant Species with Extremely Small Populations, Kunming Institute of Botany, Chinese Academy of Sciences, Kunming 650201, China

**Keywords:** *Cinnamomum chago*, kernel fatty oils, functional constipation

## Abstract

Background: *Cinnamomum chago* (*C. chago*) B.S.Sun et H.L.Zhao, a rare tree species endemic to Yunnan Province of China, produces fruit kernels that are rich in fatty oils (CCKF); however, the potential health benefits of CCKF have not been previously reported. Method: We characterized the chemical composition of CCKF and evaluated its therapeutic potential in a rat model of loperamide-induced functional constipation (FC). Results: CCKF contained an abundance of dodecanoic acid, accounting for 70.18%. CCKF alleviated constipation by increasing fecal pellet output and moisture content, enhancing small intestinal propulsion rate, and ameliorating colonic pathological damage. Additionally, CCKF modulated gastrointestinal hormone levels and enhanced the expression of ZO-1 and Occludin. Furthermore, CCKF increased gut microbial diversity, altered the *Bacillota*/*Bacteroidota* ratio, enriched potentially beneficial bacteria (including *Kineothrix*, *Roseburia*, *Lacrimispora*, and *Lawsonibacter*), and reduced *Segatella* abundance. CCKF may affect Arginine and proline metabolism, Biosynthesis of amino acids, D-Amino acid metabolism, etc. Conclusion: The present results provide a scientific basis for the potential application of CCKF as a functional food to alleviate functional constipation.

## 1. Introduction

Functional constipation (FC) is a prevalent chronic gastrointestinal disorder characterized by delayed colonic transit and impaired intestinal motility in the absence of structural or organic abnormalities [1]. Driven by shifts in modern dietary habits and sedentary lifestyles, the global incidence of FC has been rising steadily, significantly compromising patients’ quality of life and predisposing them to secondary intestinal complications. Accumulating evidence has identified intestinal dysbiosis as a pivotal pathogenic factor underlying FC [2]. Disruption of the gut microbiota can promote the proliferation of pathogenic species while suppressing beneficial commensals, alter the profile of intestinal metabolites, and ultimately induce FC [3,4].

*Cinnamomum chago* (*C. chago*) B.S.Sun et H.L.Zhao is an evergreen tree in the family *Lauraceae* and the genus *Cinnamomum* [5]. The kernel of the fruit is rich in fatty oils and possesses high nutritional value. The fatty oil content in the nuts of *C. chago* is as high as 45.66%, containing palmitic acid, oleic acid, etc. The fruit of *C. chago* can be consumed as a nut and conforms to the theory of food and medicine homology, in that it has a substance which integrates pharmacological and nutritional functions into one entity [6]. Due to their natural safety and effectiveness, food–medicine homology substances have a significant effect on the treatment and prevention of FC [7].

The positive impact of fatty oils extracted from nuts and botanical sources on intestinal well-being has been substantiated across diverse experimental frameworks. Research has found that the fatty oils from some nuts and those in plants are rich in polyunsaturated and monounsaturated fats, which can increase the diversity of gut microbiota and promote the formation of new bioactive metabolites [8,9]. Gao discovered that the kernel oil of the *Cerasus humilis* (Chinese dwarf cherry) can improve the gut microbiota balance in mice and alleviate constipation symptoms [10]. Similarly, kernel oil derived from *Torreya grandis* demonstrated therapeutic efficacy against loperamide-induced bowel dysfunction in BALB/c mice, with mechanistic insights pointing toward enhanced colonic expression of tight junction proteins (Occludin, Claudin-1, and ZO-1) alongside elevated levels of serotonin receptors (5-HT3R and 5-HT4R) [11].

Given the therapeutic potential of food–medicine homology substances against FC and the established gut-modulatory effects of plant kernel oils, *C. chago* represents a promising candidate for further investigation. However, neither the precise chemical composition of *C. chago* kernel fatty oil nor its anti-constipation efficacy and underlying mechanisms have been systematically explored. Therefore, this study extracted the kernel fatty oil of *C. chago* and conducted component analysis. The FC rat model was established using loperamide (LOP), and 16S DNA sequencing technology and metabolomics technology were employed to analyze the therapeutic effect of *C. chago* kernel fatty oil on FC, thereby providing a theoretical foundation for the development and utilization of *C. chago* as a functional food.

## 2. Materials and Methods

### 2.1. Materials and Reagents

*C. chago* was purchased from a Chinese herbal medicine market. Loperamide was procured from Sigma-Aldrich Co. LLC (St. Louis, MO, USA). Vasoactive intestinal peptide (VIP), Gastrin (GAS), somatostatin (SS), and substance P (SP) ELISA kits were acquired from Nanjing Sempega Biotechnology Co., Ltd. (Nanjing, China). Occludin (27260-1-AP), ZO-1 (217773-1-AP), and β-actin (GB15003) antibodies were procured from Proteintech Group, Inc. (Rosemont, IL, USA).

### 2.2. Physicochemical Properties and Structural Characterization of CCKF

#### 2.2.1. Extraction of Kernel Fat from *Cinnamomum chago*

The dried kernels of *Cinnamomum chago* were crushed. Then, distilled water was added at a ratio of 1:10 to the liquid, and the mixture was boiled at 70 °C for 2 h. The floating matter above the liquid was collected, which is the kernel fat oil of *C. chago* (CCKF).

#### 2.2.2. Fourier Transform Infrared Spectroscopy Analysis of CCKF

The dried CCKF sample was blended with potassium bromide at a mass ratio of 100:1 and finely pulverized. FT-IR spectra were subsequently recorded on a Bruker INVENIO S spectrometer(Bruker Optik GmbH, Ettlingen, Germany).

#### 2.2.3. Scanning Electron Microscopy Analysis of CCKF

Following dehydration, CCKF was subjected to conductive coating and then secured onto a holder. Imaging was conducted via a JEOL JSM-7610F Plus scanning electron microscope (SEM) (JEOL Ltd., Tokyo, Japan).

#### 2.2.4. GCMS of Methyl Esterified CCKF

Methylation of CCKF: The crude product was dissolved in 5 mL of petroleum ether-ether mixture (*v*/*v* = 4:3). Then, 4 mL of 0.5 mL/L NaOH-methanol solution was added, followed by shaking for 2 min and standing for 20 min. After adding 1 mL of distilled water and allowing the mixture to separate into layers, the upper layer was collected as the methylated CCKF.

Chromatographic Conditions: Separation was performed on an HP-5MS capillary column. The oven temperature program was as follows: initial temperature was held at 40 °C for 1 min, then increased to 100 °C at a rate of 13 °C/min, further increased to 110 °C at 3 °C/min and held for 1 min, then raised to 150 °C at 5 °C/min and held for 1 min, increased to 180 °C at 2 °C/min and held for 1 min, finally elevated to 250 °C at 15 °C/min and held for 2 min. The split ratio was 10:1, the injector temperature was set at 250 °C, and high-purity helium was used as the carrier gas with a flow rate of 1.2 mL/min.

Mass Spectrometry Conditions: The ionization source was electron impact (EI) with an ionization energy of 70 eV. The ion source temperature was set at 230 °C, and the quadrupole temperature was maintained at 150 °C. The mass scanning range was 30–500 amu.

### 2.3. Animal Experiments

The animal experiments were conducted with ethical approval from Henan University (HUSOM2025-030). Male Sprague Dawley (SD) rats (specific pathogen-free grade, 6–8 weeks old, 180–200 g) were purchased from Henan Sikebeisi Biotechnology Co., Ltd. (License No.: SCXK (Yu) 2020-0005, Henan, China). Before experimentation, the animals were adapted for seven days in a controlled environment (25 ± 2 °C, 40–45% humidity, 12-h light/dark photoperiod) and fed a standard diet with water ad libitum.

#### 2.3.1. Experimental Grouping

The sample size was pre-estimated using the G*Power 3.1.9.7 version. Based on our preliminary data, the effect size (Cohen’s d) was 1.45. In an independent two-tailed *t*-test with α = 0.05 and statistical power (1 − ) of 0.80, at least 9 animals were required per group. Considering the expected 15–20% attrition rate due to modeling failures or deaths, the final determination was 10 rats per group (total sample size *n* = 60).

After adaptive feeding, the animals were divided into six groups according to body weight: blank control (BC), functional constipation model (MC), positive control (PC), high-dose CCKF (HD), medium-dose CCKF (MD), and low-dose CCKF (LD). Except for the BC group, all remaining groups received loperamide (LOP) at 3 mg/kg to establish the FC model. Successful induction was verified through assessment of fecal pellet output and moisture content. Two hours after LOP administration, the PC group was given polyethylene glycol 4000 (PEG) at 3 g/kg, while the HD, MD, and LD groups were administered CCKF at 2, 1.5, and 1 g/kg, respectively, based on individual body weight. After completing a 4-week CCKF intervention, fecal samples were collected for 6 h each day for three consecutive days. Subsequently, further experiments were conducted. All animal procedures complied with ARRIVE 2.0 and the 3Rs principle to minimize suffering.

#### 2.3.2. Detection of Fecal Particle Count and Fecal Moisture Content

Three days before the end of the animal experiment, the collected fecal samples were immediately weighed, then dried, weighed again, and the moisture content was calculated.

#### 2.3.3. Determination of Small Intestinal Propulsion Rate

Before the end of the animal experiment, each rat was administered 2 mL of a 5% activated charcoal suspension by intragastric gavage. The rats were then anesthetized with chloral hydrate, and blood was collected from the abdominal aorta. Immediately after blood collection, the rats were sacrificed by exsanguination via abdominal aortic puncture under deep anesthesia, ensuring rapid and humane death before the excision of the entire small intestine. The entire small intestine was excised, laid out straight on the experimental bench, and the distance of charcoal transit was measured. The small intestine propulsion rate was calculated using the formula [12].

#### 2.3.4. Detection of Gastrointestinal Hormones in Serum

Centrifuge the blood to separate the serum sample. Following the ELISA kit instructions, detect the concentrations of VIP, GAS, SS, and SP.

#### 2.3.5. Pathological Observation of Rat Colon Tissues

Each colonic sample was fixed in 4% paraformaldehyde overnight (~24 h). The fixed tissues underwent standard histological processing: dehydration through ascending concentrations of ethanol, xylene clearing, and paraffin infiltration. Serial sections (5 μm) were stained with hematoxylin-eosin (H&E), subsequently dehydrated through a graded ethanol series, cleared in xylene, sealed with neutral gum, and inspected under an optical microscope to document histomorphological alterations.

#### 2.3.6. 16S DNA Sequencing

Qualified genomic DNA (30 ng) was combined with fusion primers to construct the PCR reaction mixture, followed by thermal amplification. The amplicons were purified with Agencourt AMPure XP magnetic beads and eluted in the supplied buffer. Indexed adapters were subsequently ligated to complete library preparation. Library fragment size and concentration were assessed on an Agilent 2100 Bioanalyzer(Agilent Technologies, Inc., Santa Clara, CA, USA). Qualified libraries were then sequenced on an Illumina platform according to insert size specifications. Raw sequencing reads were quality-filtered to retain high-quality clean reads for downstream analysis. Paired-end reads were merged into tags based on overlapping sequences, after which operational taxonomic units (OTUs) were clustered, annotated against the reference database, and subjected to species-level differential analysis. Differential abundance was assessed across the six taxonomic levels (phylum, class, order, family, genus, and species) using the Wilcoxon rank-sum test for two-group comparisons and the Kruskal–Wallis test for multiple-group comparisons. The resulting *p*-values were corrected for multiple testing using the Benjamini–Hochberg FDR method to generate q-values, which were used to evaluate statistical significance. These procedures were performed by China BGI Genomics.

#### 2.3.7. Untargeted Metabolomics Analysis of Rat Cecal Contents

Cecal content samples (n = 6 per group) were subjected to untargeted metabolomic analysis. Each aliquot (25 mg) was weighed and mixed with 800 μL of extraction solvent and 10 μL of internal standard. The subsequent method was carried out using the approach proposed by Wang et al. [13]. For metabolomic analysis, the mean abundance and standard deviation (SD) of each metabolite were calculated within each group, and the fold change (FC) between compared groups was subsequently determined. Student’s *t*-test was applied to evaluate the statistical significance of metabolite differences between groups, generating *p*-values. Multiple testing correction was performed using the Benjamini–Hochberg algorithm to derive q-values, which were then used to assess the differential significance of metabolites between the two groups. This step was completed by China BGI Genomics.

#### 2.3.8. Detection of the Expression of the Tight Junction Proteins

Total proteins from colon tissues were extracted and quantified using a BCA kit. Equal amounts of protein were separated by SDS-PAGE gel and transferred onto PVDF membranes. After blocking with 5% skim milk at room temperature for 2 h with agitation, membranes were incubated with primary antibodies (anti-ZO-1, anti-Occludin, anti-β-actin) at 4 °C overnight, and then with horseradish peroxidase-conjugated secondary antibodies at room temperature for 2 h. The blots were visualized by enhanced chemiluminescence and quantified using ImageJ.

### 2.4. Statistical Analysis

The experimental data are presented as mean ± SD. Group differences were analyzed by one-way ANOVA followed by Dunnett’s test, or Dunnett’s T3 test when variances were heterogeneous (assessed by Levene’s test), for comparisons of each group against the model group.

## 3. Results

### 3.1. Physicochemical Property Analysis of CCKF

**Figure 1:**
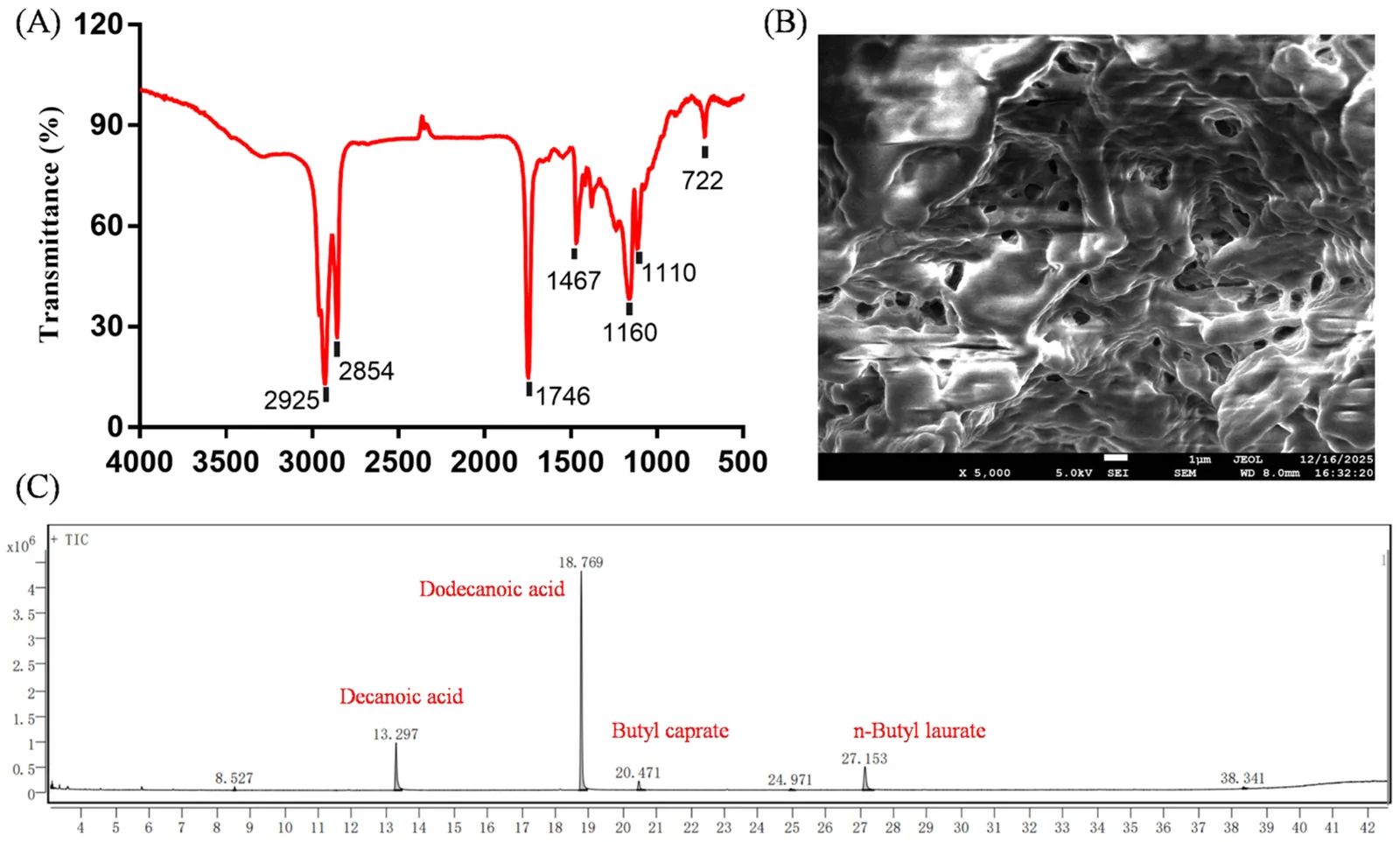
CCKF physical properties and composition analysis. (**A**) Infrared spectrogram of CCKF. (**B**) SEM image of CCKF (5000×). (**C**) TIC of CCKF.

FT-IR results (Figure 1A) showed that CCKF has prominent peaks at 2925 cm^−1^ and 2854 cm^−1^. The peak at 2925 cm^−1^ represents the asymmetric stretching vibration absorption of the saturated C-H (-CH_2_-) group, which is a characteristic absorption peak of long-chain fatty acids. The peak at 2854 cm^−1^ is the symmetrical stretching vibration absorption peak of saturated C-H (-CH_2_-), which is also a characteristic absorption peak of long-chain fatty acids. There is an absorption peak at 1746 cm^−1^. This is the C=O stretching vibration absorption peak of the ester group (-COO-), which is the most characteristic absorption peak of triacylglycerol [14], indicating that there are a large number of ester bonds in the sample. There is an absorption peak at 1467 cm^−1^, which is the bending vibration absorption peak of methylene (-CH_2_-), a characteristic absorption peak of long-chain fatty acids. However, there is no obvious C=C double bond stretching vibration absorption peak in this region (~1650 cm^−1^), indicating that the content of unsaturated fatty acids in the sample is relatively low. There are small absorption peaks at 1160 cm^−1^ and 1110 cm^−1^. These are the C-O stretching vibration absorption peaks of the ester group (-COO-), which are the characteristic absorption peaks of triacylglycerols. The shape and position of the peaks in this region are completely consistent with the typical spectra of plant oils [14].

The SEM results (Figure 1B) showed that these structural components exhibit a large number of interwoven and stacked sheet-like or block-like fat crystals, forming a continuous three-dimensional network framework. There are numerous pores of varying sizes and irregular shapes distributed among the framework. Some of these pores are interconnected, forming an open channel structure.

### 3.2. GC/MS Analysis of Methylated CCKF

The methylated CCKF was analyzed by GC/MS under the experimental conditions described above. The mass fractions of each component were determined by the area normalization method. A total of 7 components were separated from CCKF, among which 4 were identified, accounting for 93.98% of the total peak area of the chromatographic distillate (Figure 1C). The most abundant component was dodecanoic acid, accounting for 70.18% of the total peak area, followed by decanoic acid (15.26%). Butyl caprate and n-butyl laurate accounted for 1.13% and 7.41% of the total peak area, respectively. This indicated that dodecanoic acid is the main component of the CCKF.

### 3.3. The Effect of CCKF on Defecation

**Figure 2:**
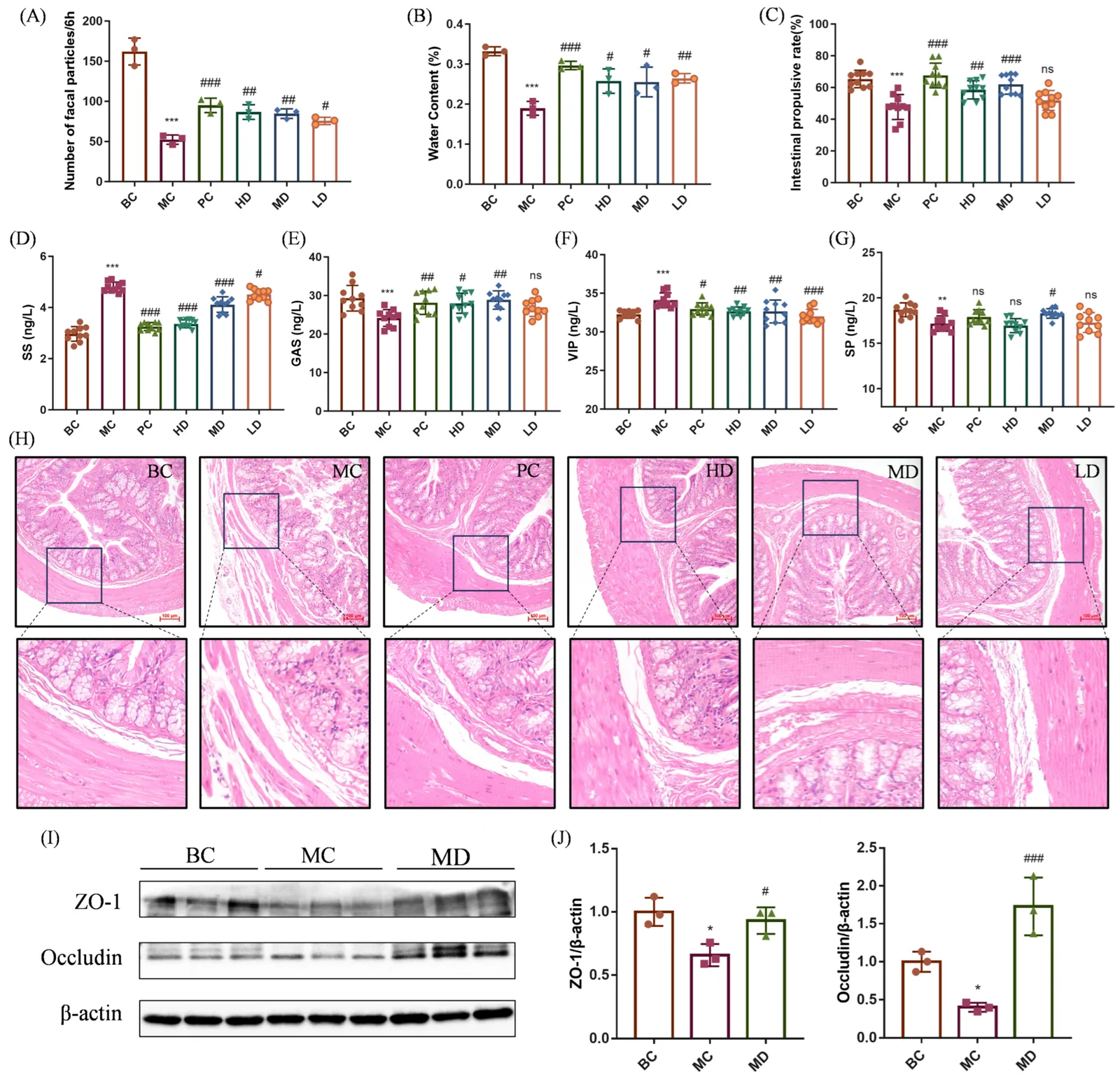
Effects of CCKF on FC rats. (**A**) Number of fecal pellets excreted within 6 h (n = 10). (**B**) Fecal water content (n = 10). (**C**) Small intestinal propulsion rate (n = 10). (**D**–**G**) Serum levels of gastrointestinal hormones (n = 10). (**H**) H&E-stained colon sections (200×). (**I**,**J**) Expression of tight junction proteins in colon tissue (n = 3). Note: Compared with the BC group, *** *p* < 0.001, ** *p* < 0.01, * *p* < 0.05; compared with the MC group, ^###^ *p* < 0.001, ^##^ *p* < 0.01, ^#^ *p* < 0.05.

The moisture content of feces and the number of fecal particles can directly assess the severity of constipation. Three days before the end of the animal experiment, we collected feces within 6 h and counted them (Figure 2A). It was found that the number of fecal particles in the MC group was significantly reduced compared with the BC group (*p* < 0.001). After administration of CCKF, the number of fecal particles significantly increased. We measured the water content in the feces (Figure 2B). It was found that the fecal water content in the MC group was significantly reduced compared with the BC group (*p* < 0.001). After administration of CCKF, the moisture content of the feces significantly increased. The results indicated that CCKF can alleviate the constipation symptoms in FC rats.

### 3.4. The Effect of CCKF on Small Intestinal Propulsion

Small intestinal propulsion rate is an important index to evaluate the peristaltic function of the small intestine, which is mainly used to quantify the movement efficiency of substances in the small intestine [12,15]. We administered 5% activated charcoal to rats by gavage and measured the migration distance of the charcoal to assess the small intestinal transit rate (Figure 2C). Compared with the BC group, the small intestinal propulsion rate in the MC group was significantly reduced (*p* < 0.001). Compared with the MC group, the intestinal propulsion rate in the PC group significantly increased (*p* < 0.001). After administering CCKF, it was found that only the HD group and the MD group could significantly increase the intestinal propulsion rate of the rats (*p* < 0.01, *p* < 0.001). However, there was no statistical difference between the LD group and the MC group. These results indicated that CCKF can increase the intestinal propulsion rate and promote defecation in FC rats.

### 3.5. The Effect of CCKF on Constipation-Related Biomarker Indexes

GAS, SS, SP, and VIP are key biomarkers in rat serum that regulate gastrointestinal motility. SP and GAS can promote gastrointestinal peristalsis, while VIP and SS can inhibit gastrointestinal peristalsis [13,16]. To investigate the modulatory effects of CCKF on gastrointestinal hormones in FC rats, serum levels of SS, GAS, VIP, and SP were determined (Figure 2D–G). The results showed that the MC group exhibited significantly increased SS (*p* < 0.001) and VIP (*p* < 0.001) levels, decreased GAS (*p* < 0.001) and SP (*p* < 0.01) levels, relative to the BC group. Compared to the MC group, the SS levels and VIP levels were reduced in the PC, HD, MD, and LD groups (*p* < 0.001, *p* < 0.001, *p* < 0.001, *p* < 0.05). Similarly, VIP levels were significantly down-regulated in the PC, HD, MD, and LD groups (*p* < 0.05, *p* < 0.01, *p* < 0.01, *p* < 0.001). Conversely, compared to the MC group, the GAS levels were increased in the PC, HD, and MD groups (*p* < 0.01, *p* < 0.05, *p* < 0.01). Similarly, the SP levels were increased in the PC and MD groups (*p* < 0.05, *p* < 0.01). The above results indicated that CCKF can restore gastrointestinal motility by regulating the levels of neurotransmitters, achieving the effect of relieving constipation. Based on the experimental findings that the MD group demonstrated superior efficacy compared to the HD and LD groups, the subsequent analysis of the gut microbiota and metabolomics was conducted on the MD group.

### 3.6. The Pathological Effects of CCKF on the Colon Tissues

Under conditions of constipation, the structure and function of the colon tissue will be damaged. Therefore, the structure and morphology of colon tissue can be directly observed by HE staining of colon sections (Figure 2H). Compared with the BC group, the colonic crypt glands and goblet cells in the MC group were damaged, and the colonic muscularis was destroyed and thinned. Compared with the MC group, the colonic crypt glands and goblet cells in the PC group and CCKF treatment group were basically restored, and the colonic muscularis was thickened with mild damage. Studies have shown that CCKF treatment can improve the pathological state of the colon and reduce colon damage.

### 3.7. Effect of CCKF on Junction Proteins in Colon Tissues

The colonic mucosal barrier relies fundamentally on intercellular tight junction proteins such as ZO-1 and Occludin [17]. Their abnormal expression or dysfunction directly affects the integrity of the colonic barrier, thereby participating in the pathogenesis of various colonic diseases. The regulatory impact of CCKF on tight junction protein expression was subsequently evaluated (Figure 2I–J. Compared to the BC group, Occludin (*p* < 0.05) and ZO-1 (*p* < 0.05) protein levels were substantially diminished; conversely, CCKF intervention significantly increased the protein levels of Occludin (*p* < 0.05) and ZO-1 (*p* < 0.001). These data demonstrate that CCKF upregulates tight junction proteins and safeguards colonic tissue barrier integrity.

### 3.8. Effects of CCKF on Gut Microbiota

**Figure 3:**
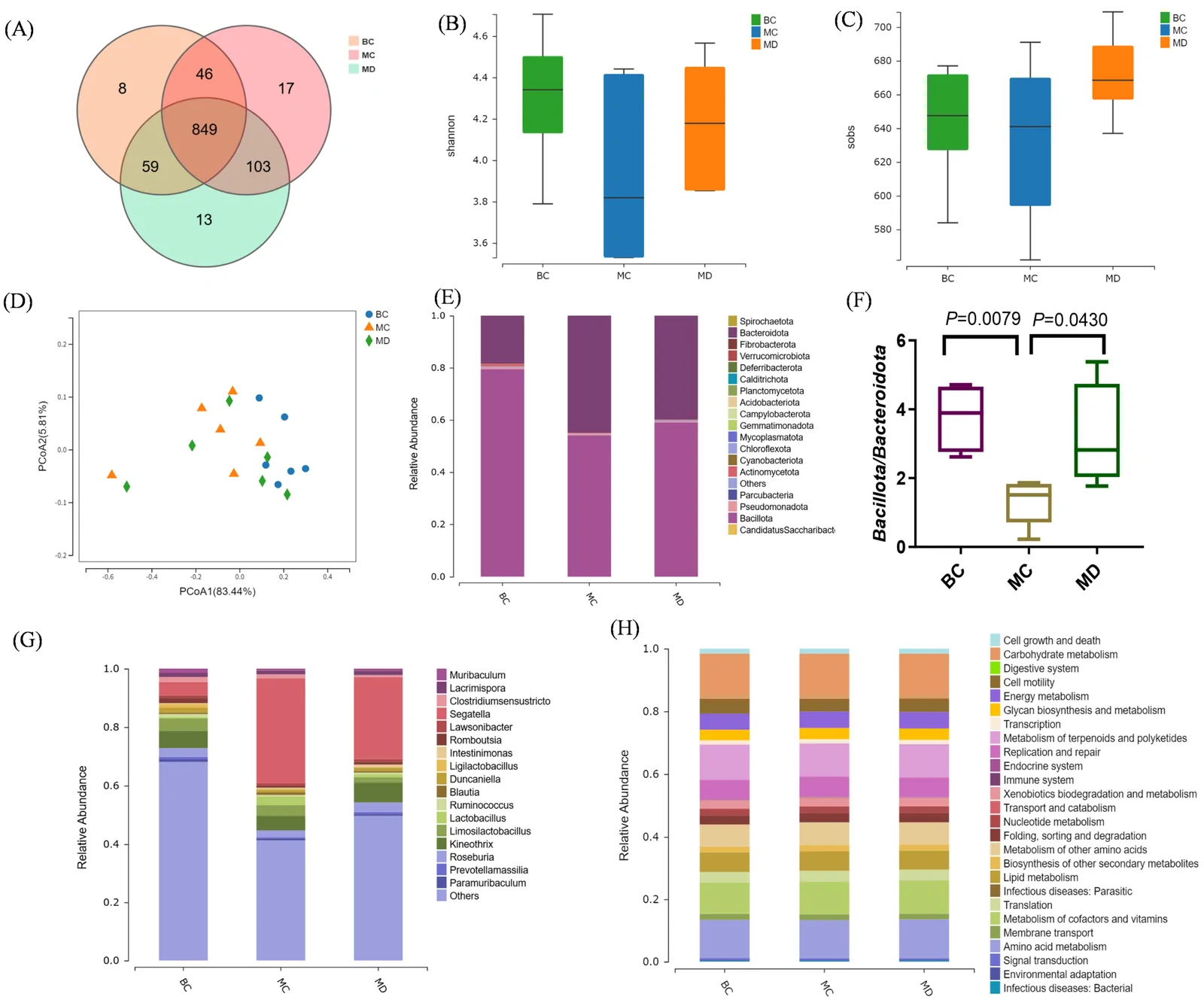
Impact of CCKF on the intestinal microbiome. (**A**) Venn diagram. (**B**,**C**) α diversity indices. (**D**) Principal coordinate analysis (PCoA). (**E**,**G**,**H**) Relative abundance of dominant microbiota at the phylum (**E**) and genus (**G**) levels. (**F**) *F*/*B* ratio. (**H**) KEGG-based functional prediction.

Gut microbiota dysbiosis plays a pivotal role in the pathogenesis of FC [18,19]. To investigate how CCKF influences gut microbiota, we performed 16S rRNA gene sequencing on rat fecal samples. Our analysis encompassed α- and β-diversity assessments, together with taxonomic comparisons at both phylum and genus levels. Additionally, all specimens underwent operational taxonomic unit (OTU) clustering followed by microbial species annotation. The results of this diagram indicate that the BC, MC, and MD groups altogether have a total of 849 OTUs (Figure 3A).

We assessed microbial community diversity through α-diversity metrics. The Shannon index reflects the comprehensive diversity of species richness and evenness in a community, with a higher value indicating greater diversity. The Sobs index represents the actual number of observed species, directly reflecting the richness of the community. Compared with the BC group, the Shannon index and Sobs index in the MC group decreased, while the Shannon index and Sobs index in the MD group increased (Figure 3B,C). This indicated that CCKF can enhance the species richness of the microbiota. In the β diversity analysis, the principal coordinate analysis (PCoA) diagram based on the Bray-Curtis distance can visually present the abundance differences of the microbial communities. In the PCoA diagrams (Figure 3D), the MC group is far away from the BC group, while the MD group is distributed around the BC group and is closer to it, indicating that the supplementation of CCKF improved the diversity of the gut microbiota in the constipated mice. This indicated that CCKF can improve the composition of the microbial community.

Based on the species annotation results, the species at the phylum level were analyzed. *Bacillota* and *Bacteroidota*, as the two dominant phyla of the gut microbiota, collectively represent over 90% of its total composition, and changes in their ratio are closely linked to the host’s intestinal health. The species annotation results revealed that the abundance of *Bacteroidota* was significantly increased, while the abundance of *Bacillota* was significantly decreased in the MC group. After CCKF treatment, the abundance of *Bacteroidota* was significantly decreased and *Bacillota* was significantly increased (Figure 3E). Under physiological conditions, the proportion of *Bacillota* relative to *Bacteroidota* (the F/B ratio) is generally maintained at a stable level. However, when intestinal microbial homeostasis is disrupted, this ratio typically exhibits a downward shift [20]. Our results showed that the F/B ratio in the MC group is lower compared with the BC group (*p* < 0.01). After CCKF treatment, the F/B ratio increases compared with the MC group (*p* < 0.05) (Figure 3F). This indicates that CCKF shifted the microbial community structure toward the profile observed in the control group. At the genus level (Figure 3G), the abundances of *Segatella*, *Lactobacillus*, and *Blautia* increased, while the abundances of *Kineothrix*, *Roseburia*, *Duncaniella*, *Lawsonibacter*, *Romboutsia*, and *Muribaculum* decreased compared to the BC group. After CCKF treatment, the abundances of *Segatella*, *Lactobacillus*, and *Blautia* decreased, while the abundances of *Kineothrix*, *Roseburia*, *Duncaniella*, *Lawsonibacter*, *Romboutsia*, and *Muribaculum* increased.

Based on the analysis of microbial metabolic functions predicted by PICRUSt2 (v2.3.0-b) software and statistically analyzed using the KEGG database (Figure 3H), the functional profile of the microbiota was primarily concentrated in Carbohydrate metabolism, Amino acid metabolism, Metabolism of terpenoids and polyketides, and Metabolism of cofactors and vitamins, etc. These findings suggested that CCKF maintains host health by regulating these metabolic functions of the gut microbiota.

### 3.9. The Effect of CCKF on the Metabolism of FC Rats

**Figure 4:**
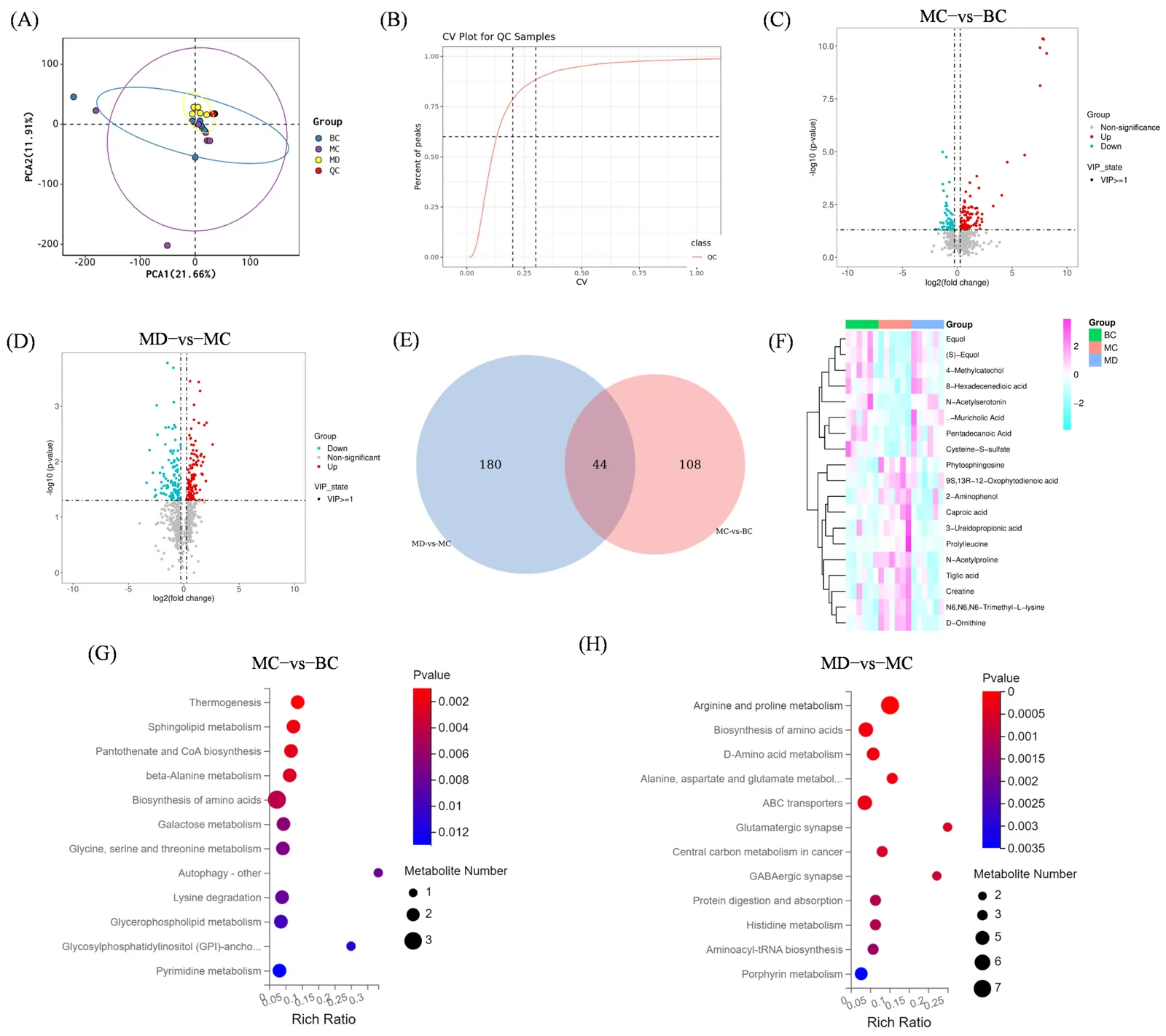
Effects of CCKF on metabolites. (**A**) PCA plot. (**B**) CV distribution of metabolites in QC samples. (**C**,**D**) Volcano plots of MC versus BC (**C**) and MD versus MC (**D**). (**E**) Venn diagram. (**F**) Clustering heatmap. (**G**,**H**) Bubble charts of metabolic pathways in MC versus BC groups (**G**) and MD versus MC groups (**H**).

Disorder of the microbiota leads to abnormal expression of metabolic products, which can affect the motility of the intestine [21,22]. To characterize the metabolome, cecal contents from six biological replicates in each of the BC, MC, and MD groups were analyzed by LC-MS/MS under both positive and negative electrospray ionization conditions. This approach collectively identified 5767 annotated metabolites across the two ionization modes. Subsequently, principal component analysis (PCA) was applied to the entire quantitative dataset to evaluate metabolic divergence among groups (Figure 4A). The PCA showed that the quality control samples (QC) were closely clustered together, indicating that the systematic error was small, the experimental repeatability was good, and the data quality was excellent. The ratio of metabolites with a coefficient of variation (CV) ≤ 30% to the total number of metabolites was 0.88 (Figure 4B), which indicated that the detection data of this experiment were qualified.

Significantly altered metabolites were identified according to the following thresholds: variable importance projection (VIP) ≥ 1, fold change (FC) ≥ 1.2, and q < 0.05. Metabolites with low credibility were excluded. The screening results are presented through a volcano graph (Figure 4C,D). Compared with the BC group, the MC group displayed 96 upregulated metabolites and 55 downregulated metabolites in the MC group. Meanwhile, the MD group displayed 118 upregulated and 106 downregulated metabolites when contrasted with the MC group. The results of Venn visualization showed that 44 metabolites were common in the BC, MC, and MD groups. Visual analysis of key differential metabolites was conducted and clustering heat maps were drawn (Figure 4F). In the clustering heat map, the columns represent the samples, the rows represent the different metabolites, and the shade of the color indicates the expression level.

To further analyze the metabolic pathways enriched by these metabolites, KEGG enrichment analysis was carried out on the differentially abundant metabolites. Based on the *p* values, the top 12 metabolic pathways were listed (Figure 4G,H). The differentially expressed metabolites in the MC versus BC group were mainly enriched in pathways such as thermogenesis, sphingolipid metabolism, pantothenate and CoA biosynthesis, beta-Alanine metabolism, biosynthesis of amino acids, galactose metabolism, glycine, serine and threonine metabolism, autophagy, lysine degradation, etc. After administration of CCKF, these differential metabolites were mainly enriched in Arginine and proline metabolism, Biosynthesis of amino acids, D-Amino acid metabolism, Alanine, aspartate and glutamate metabolism, ABC transporters, Glutamatergic synapse, Central carbon metabolism in cancer, GABAergic synapse, Protein digestion and absorption, Histidine metabolism, etc. The above results indicated that the intervention effect of CCKF mainly lies in its profound regulation of the amino acid metabolism network (particularly the Arginine and proline metabolism), significantly influencing the neurotransmitter system (glutamatergic and GABAergic synapses).

### 3.10. Association Analysis of Metabolites and Intestinal Microbiota

**Figure 5:**
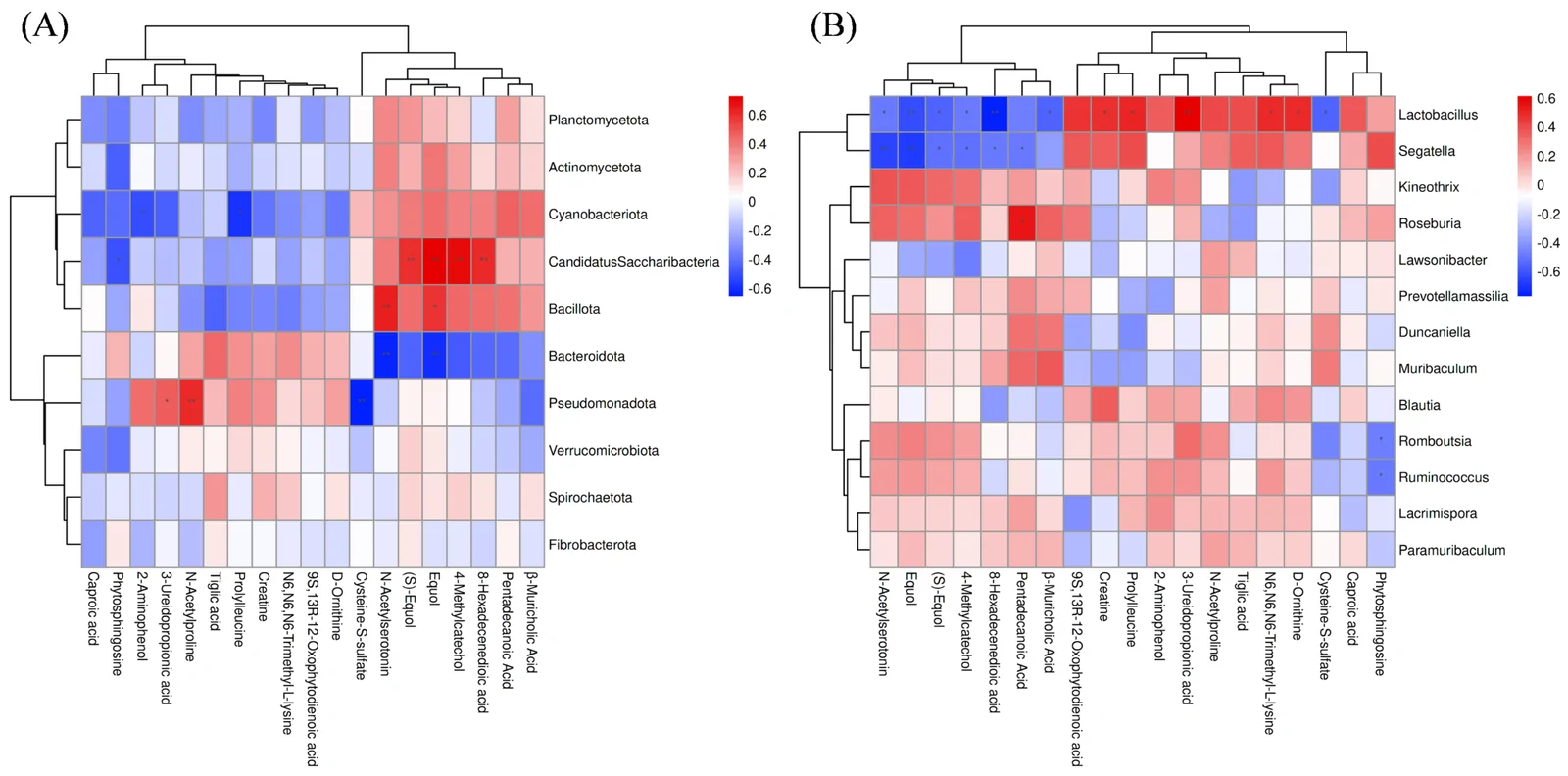
Heat map of correlations between gut microbiota and differential metabolites. (**A**) Phylum. (**B**) Genus. Different colors represent positive correlation (red) and negative correlation (blue). * indicates *p* < 0.05, and ** indicates *p* < 0.01.

We conducted a Spearman correlation analysis between the differential metabolites and the key bacterial populations. At the phylum level (Figure 5A), we selected the top 10 most abundant microbial groups for correlation analysis with differential metabolites. The results revealed that *Candidatus Sacchari bacteria*, *Bacillota*, *Pseudomonadota*, and *Bacteroidota* exhibited strong correlations with certain metabolites. At the genus level (Figure 5B), we conducted an association analysis between the top 13 most abundant bacterial groups and the differential metabolites. The results showed that *Lactobacillus* and *Segatella* had a correlation with the metabolites. These findings suggest a potential metabolic interplay between the gut microbiota and host metabolism following CCKF intervention, although the directionality and causality of these relationships remain to be determined.

## 4. Discussion

The fruit of *C. chago* is a kind of edible nut, and its kernel contains a large amount of fatty oil. The GC/MS results showed that CCKF contained a large amount of saturated fatty acids, such as dodecanoic acid, decanoic acid, n-butyl laurate, and butyl caprate. Among them, dodecanoic acid and decanoic acid were the main components, with only a small amount of n-butyl oleate and butyl caprate. Dodecanoic acid is also known as lauric acid. It has been reported that lauric acid can inhibit LPS-induced intestinal flora imbalance and alter the composition of the intestinal microbiota [23]. Decanoic acid can significantly improve the composition of the gut microbiota and enhance the intestinal barrier function [24]. Given that CCKF is abundant in such bioactive fatty acids, it is reasonable to hypothesize that this oil may ameliorate FC through modulation of the gut microbiota and intestinal metabolites. To test this hypothesis, the present research established a LOP-induced FC rat model and employed 16S DNA sequencing coupled with untargeted metabolomics to systematically investigate the effects of kernel oil of *C. chago* on gut microbial and metabolic profiles, thereby elucidating its underlying mechanism of action against FC.

Animal experiments revealed that CCKF effectively increased the number of fecal particles and fecal moisture content, enhanced small intestinal propulsion, and alleviated constipation symptoms. Meanwhile, CCKF increased SP and GAS levels, decreased VIP and SS levels, ameliorated colonic histopathological changes, up-regulated tight junction protein expression (ZO-1 and Occludin), attenuated intestinal barrier injury, and preserved colonic barrier integrity. These findings collectively indicate that CCKF exerts a protective effect against FC.

16S DNA gene sequencing revealed that CCKF significantly increased gut microbial diversity and optimized community structure. At the phylum level, CCKF can increase the abundance of *Bacillota* and decrease the abundance of *Bacteroidota*. The phylum *Bacillota* predominantly comprises Gram-positive bacteria and represents the highest proportion in the gut microbiota, influencing digestive, immune, and metabolic balance through the production of short-chain fatty acids (SCFAs). The abundance of *Bacteroidota* significantly increases in severe FC cases, which is a characteristic that distinguishes severe FC [25]. Excessive *Bacteroidota* proliferation could impair mucin’s defensive and lubricating capacity, which in turn results in a depleted mucus layer and dysfunctional intestinal barriers [26,27,28]. This indicated that supplementing CCKF can regulate the gut microbiota and maintain stability. The genus-level annotation results showed that *Segatella* (Phylum *Bacteroidota*) was the dominant genus. Our results showed that the level of *Segatella* in the MC group was significantly increased. After administration of CCKF, the abundance of *Segatella* decreased. Previous studies have indicated that it can regulate the TLR4/NF-κB signaling pathway to mediate the synergistic effect of inflammatory response and metabolic toxicity [29]. Studies have shown that CCKF can regulate inflammatory responses by regulating *Segatella*, affect the metabolism of metabolites in the body, and thereby influence the health status of the organism [30]. In addition, among the results of the species annotation, the majority of the bacteria belong to the *Bacillota* phylum, such as *Kineothrix*, *Lactobacillus*, *Roseburia*, *Lacrimispora*, *Blautia*, *Lawsonibacter*, and *Romboutsia*, with *Kineothrix* exhibiting the highest relative abundance. CCKF can increase the abundance of *Kineothrix*, *Roseburia*, *Lacrimispora*, *Lawsonibacter*, and *Romboutsia*, while reducing the abundance of *Lactobacillus* and *Blautia*. *Kineothrix*, *Roseburia*, *Lacrimispora*, and *Lawsonibacter* are all closely related to intestinal health. During their metabolic processes, they can ferment carbohydrates to produce SCFAs, which is crucial for maintaining the integrity of the intestinal barrier and regulating the host’s immune response [31,32,33,34]. In addition, it has been reported that *Kineothrix* can also alleviate liver damage by restoring lipid metabolism, thereby reducing intestinal inflammation [35]. *Roseburia* is a marker of a protective microorganism in most studies and may serve as a biomarker for a variety of diseases [36,37]. *Romboutsia* can promote the metabolism of lipids into long-chain fatty acids by producing secondary bile salts, which are then fermented to produce SCFAs to provide energy [38]. It is worth noting that *Lactobacillus* and *Blautia* are probiotic bacteria that can enhance intestinal barrier function and relieve constipation in multiple ways [39,40,41]. However, in our results, the abundance of *Lactobacillus* increased in the MC group, but decreased after administration of CCKF. There are also reports indicating that the number of *Lactobacillus* significantly increased in the constipation model [42,43]. This may be attributed to alterations in specific species within the genera *Lactobacillus* and *Blautia*, resulting in an apparent increase at the genus level; it may also be related to the duration and concentration of loperamide administration, as well as the mouse strain. Therefore, further exploration is needed to understand the impact of *Blautia* and *Lactobacillus* on FC.

Metabolomics analysis revealed that the metabolites after CCKF treatment were mainly concentrated in Arginine and proline metabolism, Biosynthesis of amino acids, D-Amino acid metabolism, Alanine, aspartate and glutamate metabolism, ABC transporters, etc. In the Arginine and proline metabolism pathway, the Creatine (Cr) level increased in the MC group and decreased after the administration of CCKF. Cr is an amino acid derivative that has been shown to have antioxidant, anti-inflammatory, and bioenergy benefits against various diseases. It strengthens barrier integrity by maintaining ATP levels in intestinal epithelial cells, protecting cells from oxidative stress and inflammatory mediators, and promoting tight junction protein expression [44,45]. Creatine transporter 1 (CrT1) is the main channel through which creatine enters cells. Under inflammatory conditions, the expression of CrT1 is downregulated, resulting in the inability of intestinal epithelial cells to absorb sufficient creatine [46]. After administration of CCKF, the level of creatine in intestinal metabolites decreased. This might be due to more creatine being absorbed, improving the intestinal barrier function and reducing colonic tissue damage. In the Biosynthesis of amino acids pathway, the level of Cysteine-S-sulfate decreased in the MC group and increased after administration of CCKF. Cysteine-S-sulfate is a structural analogue of glutamate and can act as an agonist for N-methyl-D-aspartate receptors (NMDA-R) [47]. The activation of NMDA-R in intestinal neurons is associated with the regulation of intestinal motility, influencing the development of constipation.

While the observed correlations between specific bacterial taxa and differential metabolites support the hypothesis that CCKF modulates gut microbiota structure and function, these associative patterns do not establish causality. The observed relationships may reflect microbiota-driven metabolic shifts, metabolite-mediated microbial selection, or independent host responses to CCKF, and thus should be interpreted with caution. Furthermore, given the well-documented interspecies differences in gut microbiota composition, gastrointestinal physiology, and host–microbe interactions, extrapolation of these murine findings to human populations requires careful validation. Future studies employing gnotobiotic animal models, fecal microbiota transplantation, and targeted metabolite or bacterial supplementation will be essential to dissect the causal interplay between microbiota and host metabolism and to confirm the therapeutic relevance of CCKF in clinical settings.

*C. chago*, a unique and rare plant resource in China, is characterized by a rich nutritional profile and distinctive bioactive constituents. It contains a large amount of medium-chain fatty acids (such as lauric acid and caprylic acid), which are extremely rare among the Cinnamomaceae plants. Studies have reported that the polysaccharides of *C. chago* can activate the Nrf2/NQO1 signaling pathway in the liver and protect the liver [48]. Our research findings indicated that the kernel fat oil of *C. chago*, as a unique lipid component of plants in the *Lauraceae* family, has a favorable regulatory effect on the gut microbiota and intestinal barrier. These research findings aim to establish a theoretical framework for *C. chago*’s medicinal potential and advance the innovation of plant-based anti-constipation agents.

## 5. Conclusions

This study demonstrated that CCKF could alleviate constipation symptoms in LOP-induced FC rats, reduce colonic damage, enhance the expression of tight junction proteins (ZO-1/Occludin), and protect the intestinal barrier. In addition, CCKF can also reshape the gut microbiota structure, increase the ratio of *Bacillota/Bacteroidota*, enhance the abundance of *Kineothrix*, *Roseburia*, *Lacrimispora,* and *Lawsonibacter*, and reduce the abundance of *Segatella*. CCKF affects Arginine and proline metabolism, Amino acid biosynthesis, and D-amino acid metabolism. In conclusion, this study provides a direction for further exploration of the mechanism of CCKF to treat constipation.

## Data Availability

All data supporting the findings of this study are available within the article.
